# Cone Beam Computed Tomography Image Quality Improvement Using a Deep Convolutional Neural Network

**DOI:** 10.7759/cureus.2548

**Published:** 2018-04-29

**Authors:** Satoshi Kida, Takahiro Nakamoto, Masahiro Nakano, Kanabu Nawa, Akihiro Haga, Jun'ichi Kotoku, Hideomi Yamashita, Keiichi Nakagawa

**Affiliations:** 1 Radiology, The University of Tokyo Hospital; 2 Radiation Oncology, The Cancer Institute Hospital, Japanese Foundation for Cancer Research; 3 Radiology, Biomedical Sciences, Tokushima University Graduate School; 4 Graduate School of Medical Care and Technology, Teikyo University

**Keywords:** cone beam ct, planning ct, deep learning, convolutional neural network, image quality, deformable image registration

## Abstract

Introduction

Cone beam computed tomography (CBCT) plays an important role in image-guided radiation therapy (IGRT), while having disadvantages of severe shading artifact caused by the reconstruction using scatter contaminated and truncated projections. The purpose of this study is to develop a deep convolutional neural network (DCNN) method for improving CBCT image quality.

Methods

CBCT and planning computed tomography (pCT) image pairs from 20 prostate cancer patients were selected. Subsequently, each pCT volume was pre-aligned to the corresponding CBCT volume by image registration, thereby leading to registered pCT data (pCT_r_). Next, a 39-layer DCNN model was trained to learn a direct mapping from the CBCT to the corresponding pCT_r _images. The trained model was applied to a new CBCT data set to obtain improved CBCT (i-CBCT) images. The resulting i-CBCT images were compared to pCT_r_ using the spatial non-uniformity (SNU), the peak-signal-to-noise ratio (PSNR) and the structural similarity index measure (SSIM).

Results

The image quality of the i-CBCT has shown a substantial improvement on spatial uniformity compared to that of the original CBCT, and a significant improvement on the PSNR and the SSIM compared to that of the original CBCT and the enhanced CBCT by the existing pCT-based correction method.

Conclusion

We have developed a DCNN method for improving CBCT image quality. The proposed method may be directly applicable to CBCT images acquired by any commercial CBCT scanner.

## Introduction

Cone beam computed tomography (CBCT) plays an important role in image-guided radiation therapy (IGRT), while having disadvantages of severe shading artifact caused by the reconstruction using scatter contaminated and truncated projections [1-4]. Scatter correction for the CBCT has been extensively studied so far [3]. To remove scattered photons from the projection images, some attempts were made by changing the irradiating arrangement [5] or by inserting an anti-scatter grid [6]. A more effective correction method was proposed by directly estimating the scatter component using a lattice-shaped lead beam stopper positioned in front of the X-ray tube [7], thus partly blocking the X-ray beams. The scatter component may be therefore estimated from the pixel values in the X-ray blocked region on the acquired projection image. Several methods for estimating the scatter component without modifying the X-ray hardware have been proposed by way of either analytical [8, 9] or Monte Carlo modeling approach [10, 11]). The analytical modeling approach has been widely employed for the scatter correction in the current clinical CBCT systems, where the detected image was given by the convolution integral of a direct transmission component and a point spread function. Then, the direct transmission component can be restored by deconvolution [9]. An iterative method for more accurate scatter correction was also proposed, where the deconvolution for the scattering correction was repeatedly performed until the voxel values of the reconstructed image converged [12].

Knowing the existing correction techniques mentioned above, the CBCT image quality may still have room for improvement [2]. For example, planning computed tomography (pCT) was used as prior information for further improvement since the image quality of the pCT was higher than that of CBCT [13-15]. A known problem of this technique was that the scatter correction may generate false structures in the CBCT images by considering anatomical structures appeared only in the pCT images. In order to solve the issue, low spatial-frequency scatter correction was performed without considering high-frequency components, based on the fact that the anatomical structures had high-frequency components [13-15]. However, some image errors still emerge in the area where pCT and CBCT have different anatomies, causing contrast deterioration and tissue alteration [14]. In order to suppress the false structures in the CBCT images, a method using sparse sampled low-frequency components has been proposed [15], but high-frequency artifacts such as streaks still cannot be removed.

In the meantime, several deep learning algorithms were proposed to improve image quality using different network models [16-18]. In particular, deep convolutional neural networks (DCNN) are powerful techniques for feature extraction and were applied to image denoising, deblurring and super-resolution [17, 18]. In the field of medical image processing, the DCNN was already applied to lesion detection [19] and image segmentation [20]; however, there are few studies for image quality improvement.

In this work, we propose a DCNN method for producing high-quality CBCT images. So far, magnetic resonance imaging (MRI)-based synthetic computed tomography (CT) generation was reported by applying a DCNN to learn a direct mapping from MRI images to their corresponding CT images [21]. Low-dose CT image restoration was also realized by applying a DCNN to learn mapping from low-dose CT images to corresponding normal-dose CT images [22]. We thus aim to produce high-quality CBCT images by applying a DCNN to learn a direct mapping from the original CBCT images to their corresponding pCT images. To our knowledge, this is the first report proposing a DCNN method for improving CBCT image quality.

## Materials and methods

Data acquisition and image processing

In this study, CBCT and pCT image pairs from 20 prostate cancer patients were used who underwent stereotactic radiotherapy with an Elekta Synergy linear accelerator (Elekta AB, Stockholm, Sweden). The pCT images had a matrix size of 512 by 512 on the axial plane with a pixel size of 1.074 mm by 1.074 mm, and a slice thickness of 1 mm. Five CBCT data sets per patient were obtained during the course of the treatment using a kV on-board imager (XVI) with a tube voltage 120 kV, an exposure of 350 mAs and were output to match the resolution and the slice thickness of the pCT images automatically using XVI.

**Figure 1:**
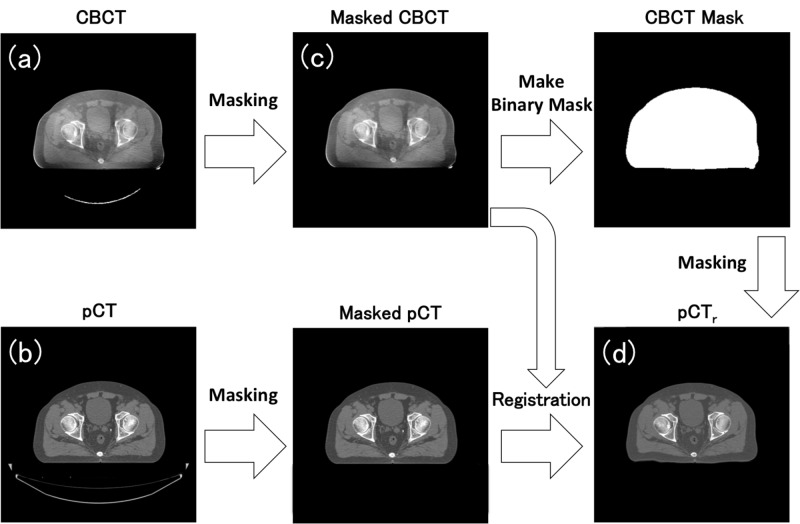
Workflow of the preprocessing to create a pair image of CBCT and pCT to be learned by DCNN. (a) and (b) show original CBCT and pCT before masking and DIR, whereas (c) shows masked CBCT and (d) shows pCT_r_ image masked by a binary mask image of CBCT. (c) and (d) depict an example of the 2D image pair to be learned by DCNN. CBCT: Cone beam computed tomography; pCT: Planning computed tomography; pCT_r_: Registered planning computed tomography; DIR: Deformable image registration; DCNN: Deep convolutional neural network.

Workflow of the preprocessing to create a pair image of CBCT and pCT to be learned by DCNN was shown in Figure 1. To avoid any adverse impact of non-anatomical structures on a CBCT to pCT registration and a model training procedure, binary masks were created to separate the pelvic region from non-anatomical regions based on each pair of the CBCT and pCT images. These masks were achieved by applying the Otsu auto-thresholding method on each CBCT and pCT image, and the voxel values outside the mask region were entirely replaced with a Hounsfield Unit (HU) of -1000. Then, the masked pCT volume data for each patient were three-dimensionally pre-aligned to each of the five masked CBCT images by rigid and deformable image registration (DIR) using an open source software, Elastix [23], resulting in a registered pCT hereinafter referred to as pCT_r_. The binary mask image of each CBCT was applied to each pCT_r_, and the voxel values outside the mask region were replaced with an HU of -1000.

DCNN model

**Figure 2:**
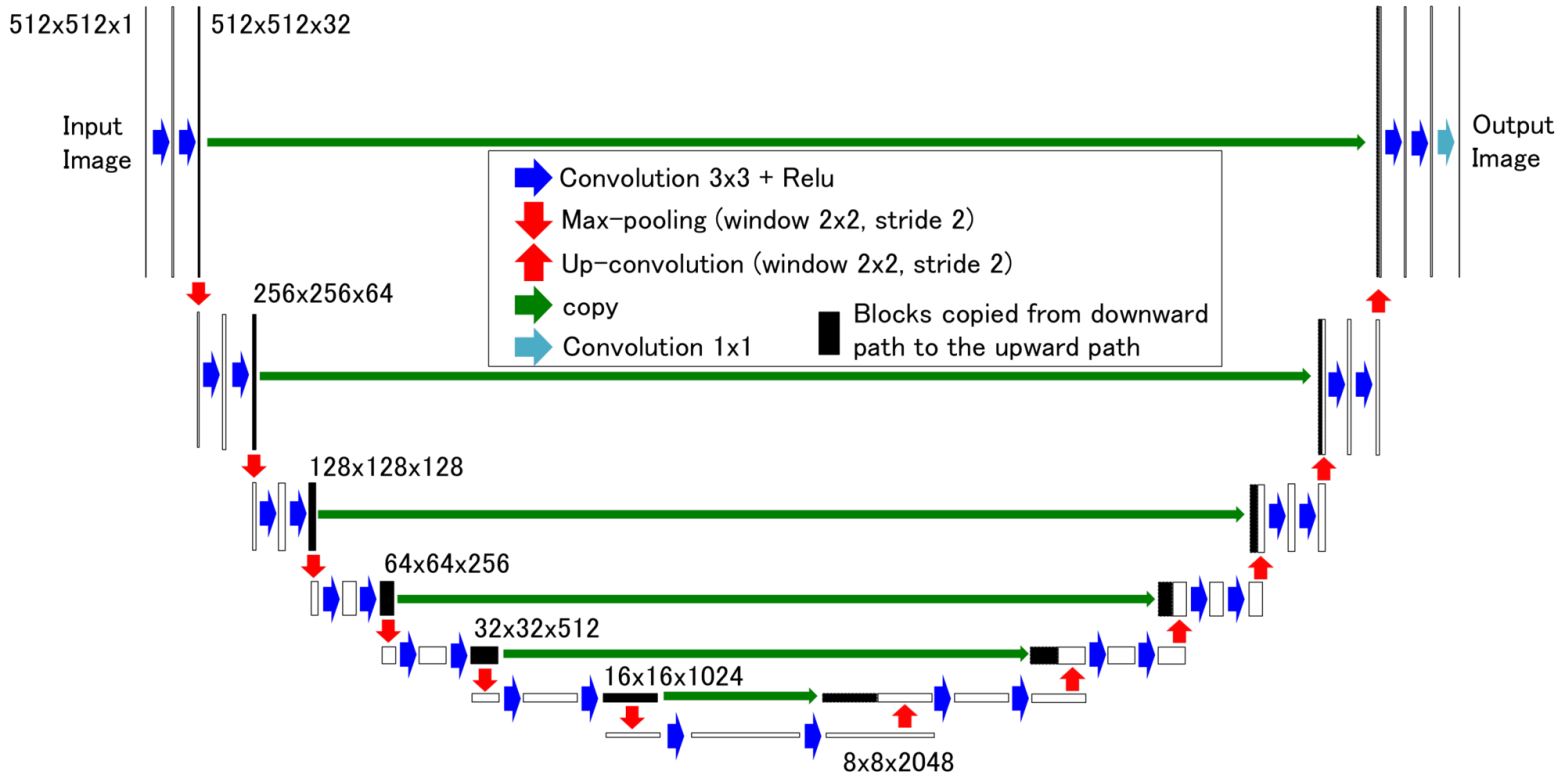
Deep convolutional neural network (DCNN) model with U-net architecture used in this study. The model consisted of two parts: a downward encoding part (left half) and an upward decoding part (right half), where 3 x 3 convolution layer with a rectified linear unit (Relu) as the activation function (blue) and Max pooling/unpooling (red) with a 2 x 2 window and stride 2 were repeatedly applied. Blocks were copied from the downward path to the upward path (green). 1 x 1 convolution was performed to generate the output image (light blue). The 2D image size and the number of channels of the feature map from each layer were appropriately provided.

U-net convolutional network architecture was recently developed for fast and precise object segmentation in a 2D image [24]. We modified the network as shown in Figure 2. The DCNN we designed has 39 layers in total and has approximately 125.8 million parameters. These parameters (θ) are optimized by minimizing a loss error between the predicted images and the corresponding ground truth pCT_r_ images Y. Given a set of CBCT images and their corresponding pCT_r_ images, the mean absolute error (MAE) as the loss function is defined as follows:

$MAE\left ( \Theta \right )= \frac{1}{N}\frac{1}{M}\sum_{i= 1}^{N}\sum_{j= 1}^{M}\left | Y_{i, j} - P\left ( X_{i, j};\Theta \right ) \right |,$

where N is the number of training 2D images and M is the total number of pixel per 2D image. The MAE loss function facilitates robust machine learning of outliers caused by noises, artifacts and misalignment between the training CBCT and pCT_r_ data. The proposed DCNN model was implemented in a graphics processing unit (GPU) using the publicly available Keras package [25]. The model was trained using the Adam stochastic optimization method [26]. The learning rate $\alpha$ was set to 0.001 and the exponential decay rates $\beta _{1}$ and $\beta _{2}$ were set to 0.9 and 0.999, respectively, which were the same values as the default setting suggested in the original paper [26]. A batch size, which is a subset size of training samples for each iteration of the optimization, was set to 10 as permitted by the GPU card. The calculation time for training was nearly a day using a single NVIDIA Titan X GPU with 3584 cores and 12 GB memory.

The performance of the proposed DCNN was evaluated using a fivefold cross-validation procedure. Twenty cases were randomly divided into five groups of the same size, whilst five pairs of the CBCT and pCT_r_ images per patient were not divided. One group was retained as test data, and the remaining four groups were used for training. Each of the CBCT and pCT_r_ images had approximately 180 slices in all the 20 cases. We employed 16 training cases of five image pairs per patient; and therefore, the total number of training slices was approximately 14400. If the MAE of the test data was not updated more than 20 epochs while the MAE of the training data kept falling, it was regarded as over-learning and the training was stopped. Finally, the test CBCT images were fed into the trained DCNN model to obtain the improved CBCT (i-CBCT) images.

Evaluation method

To evaluate the performance of the proposed method, the comparison to another existing planning CT-based correction method (not in the projection domain but in the image domain) [22] was made. The existing method has demonstrated great successes on enhancing CBCT image qualities, and is considered as a benchmark method in clinical studies [27]. The corrected CBCT by the existing method hereinafter referred to as “enhanced CBCT”.

In this study, pCT_r_ was considered as ground truth, and image quality was evaluated on selected slices in terms of spatial nonuniformity (SNU) and mean pixel values. SNU was defined as follows [15]:

$SNU= \bar{\mu }_{max} - \bar{\mu }_{min}$

where  $\bar{\mu }$ denotes a mean pixel value inside a square region of interest (ROI) with 10 by 10 pixels. $\bar{\mu }_{max}$ and $\bar{\mu }_{min}$ are the maximum and the minimum mean pixel values among five square ROIs having 10 by 10 pixels, which were arbitrary positioned in the regions of the same soft tissue area (fat or muscle) in distant locations. The pixel value consistency of the same soft tissue in distant locations means the spatial uniformity of the entire image.

The mean pixel value among the five ROIs giving the maximum difference between pCT_r_ and original CBCT images (defined as ROI_m_) was also used as an index of image quality improvement. For this evaluation, fat ROIs and muscle ROIs were used for patients 1-10 and 11-20, respectively. The root mean square differences (RMSD) of the SNU and the mean pixel value of ROI_m_ between pCT_r_ and CBCT were calculated.

The peak-signal-to-noise-ratio (PSNR) was measured to capture the reduction of noise and the structural similarity index measure (SSIM), which is one of the human visual system-based metrics, and to evaluate different attributes such as luminance, contrast and structure comprehensively. The PSNR and the SSIM of CBCTs were measured based on pCT_r_ in the five ROIs used in calculation of SNU for each patient and resulting five values were averaged. Suppose $x$ are CBCT images (original CBCT, enhanced CBCT and i-CBCT) and $y$ are pCT_r_ images, we defined PSNR and SSIM as follows:

$PSNR(x, y)= 20\cdot log_{10}(MAX) - 10\cdot log_{10}(\frac{1}{MN}\sum_{i=0}^{M-1}\sum_{j=0}^{N-1}\left [x(i,j)-y(i,j) \right ]^{2})$

where $MAX= 65535$, $M=10$, $N=10$.

$SSIM(x,y)= \frac{(2\mu _{x}\mu _{y}+C_{1})(2\sigma _{xy}+C_{2})}{(\mu {_{x}}^{2}+\mu {_{y}}^{2}+C_{1})(\sigma {_{x}}^{2}+\sigma {_{y}}^{2}+C_{2})}$

where $C_{1} = (K_{1}L)^{2}$, $C_{2}= (K_{2}L)^{2}$,

$\mu _{x}=\frac{1}{MN}\sum_{i=0}^{M-1}\sum_{j=0}^{N-1}x(i,j)$,

$\sigma _{x}= \sqrt{\frac{1}{MN-1}\sum_{i=0}^{M-1}\sum_{j=0}^{N-1}(x(i,j)-\mu _{x})^{2}}$,

$\sigma _{xy}= \frac{1}{MN-1}\sum_{i=0}^{M-1}\sum_{j=0}^{N-1}(x(i,j)-\mu _{x})(y(i,j)-\mu _{y})$,

where $L=3000$, $K_{1}=0.01$, $K_{2}=0.03$, $M=10$, $N=10$.

In order to highlight the differences of the images (pCT_r_, original CBCT, enhanced CBCT and i-CBCT), differences between some images were calculated.

## Results

**Figure 3:**
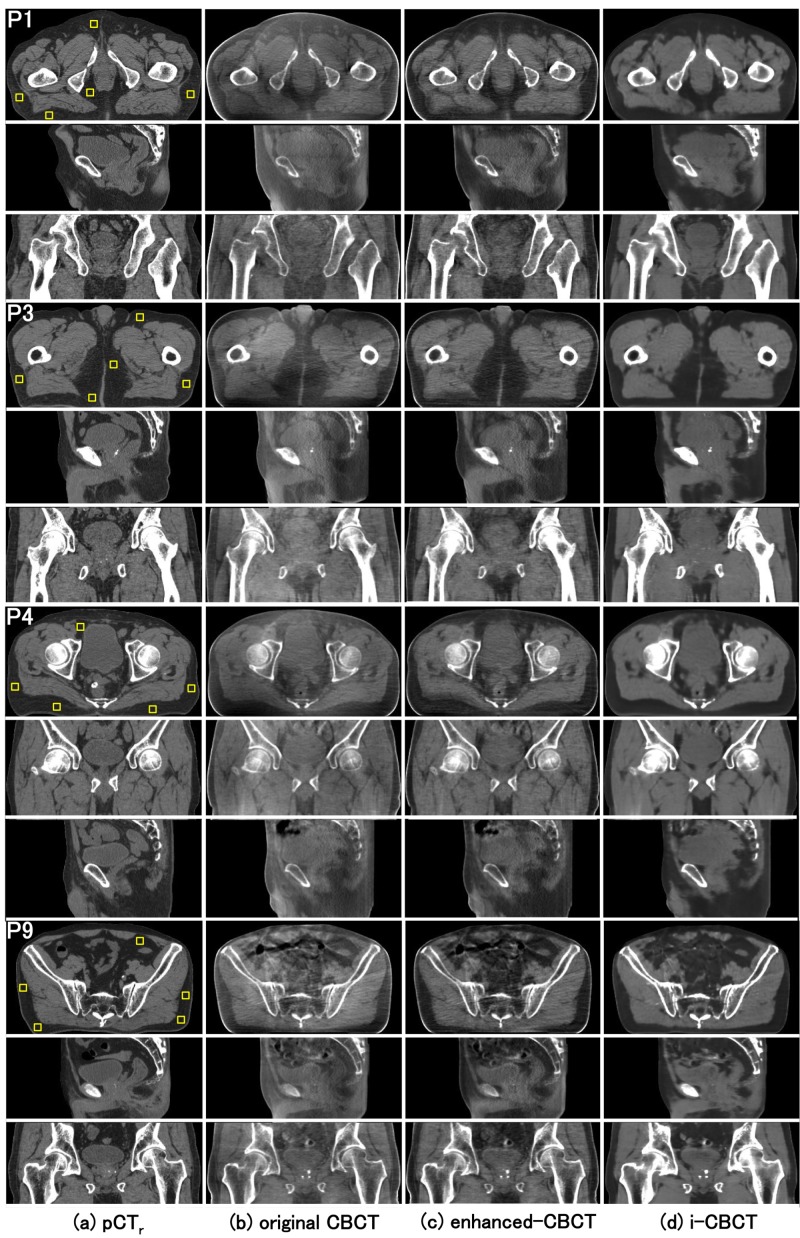
Comparison of the image qualities for representative four cases (patient 1, 3, 4, 9). For each patient, the images in the top, middle, and bottom row are axial, sagittal and coronal views, respectively. Columns (a), (b), (c) and (d) are pCT_r_, original CBCT, enhanced CBCT and i-CBCT, respectively. The yellow squares placed on the axial view of the column (a) were selected ROIs for the SNU calculation. The ROIs were set on the fat region for each patient. Display window range was set to (-400, 100) HU for the original CBCT and (-300, 200) HU for the pCT_r_, the enhanced CBCT and the i-CBCT. pCT_r_: Registered planning computed tomography; original CBCT: Original cone beam computed tomography; enhanced-CBCT: Enhanced cone beam computed tomography; i-CBCT: Improved cone beam computed tomography; SNU: Spatial nonuniformity; ROI: Region of interest.

**Figure 4:**
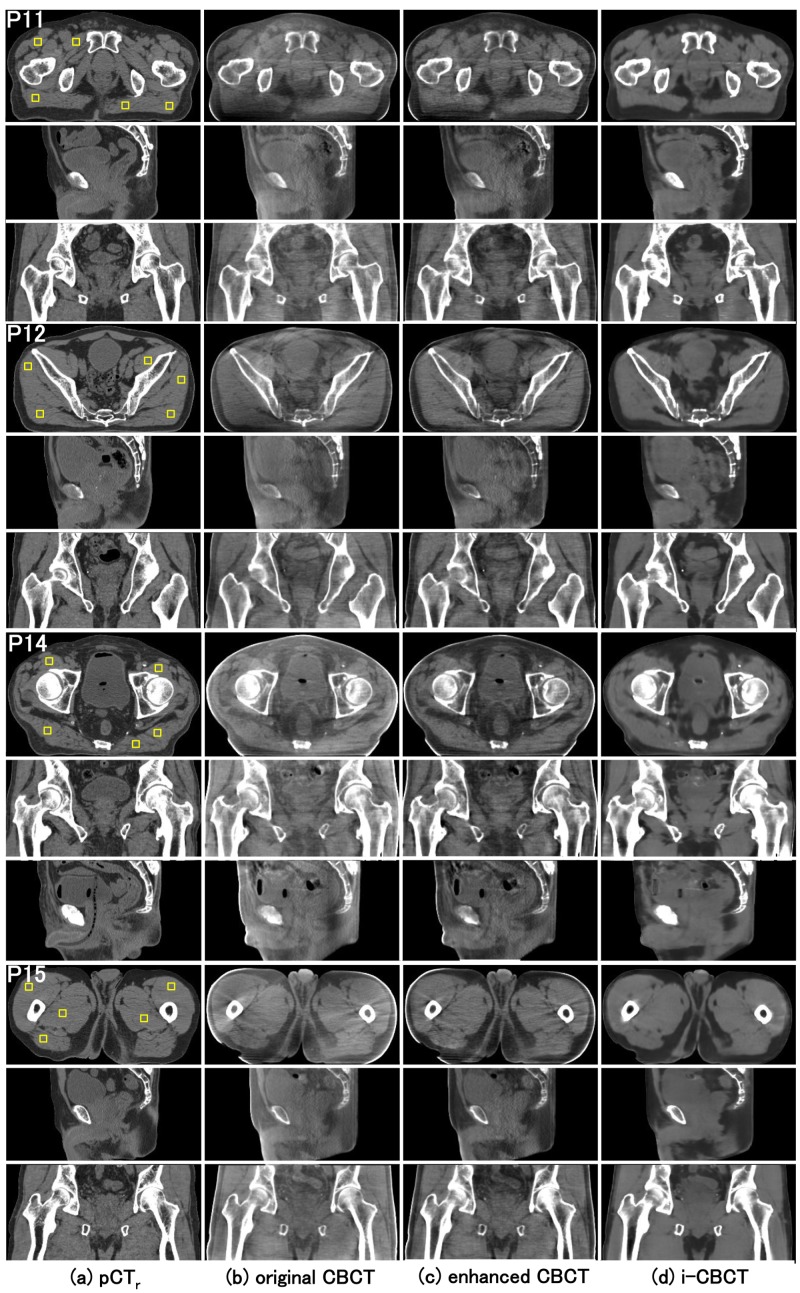
Comparison of  the image qualities for another four cases (patient 11, 12, 14, 15). For each patient, the images in the top, middle, and bottom row are axial, sagittal and coronal views, respectively. Columns (a), (b), (c) and (d) are pCT_r_, original CBCT, enhanced CBCT and i-CBCT, respectively. The yellow squares placed on the axial view of the column (a) were selected ROIs for the SNU calculation. The ROIs were set on the muscle region for each patient. Display window range was set to (-400, 100) HU for the original CBCT and (-300, 200) HU for the pCT_r_, the enhanced CBCT and the i-CBCT. pCT_r_; Registered planning computed tomography; original CBCT: Original cone beam computed tomography; enhanced-CBCT: Enhanced cone beam computed tomography; i-CBCT: Improved cone beam computed tomography; ROI: Region of interest; SNU: Spatial nonuniformity.

Once the model had been trained, approximately 20 seconds were required to convert from 180 slices of a new patient CBCT data set to corresponding i-CBCT images. The axial, sagittal, and coronal slices of eight patient cases are shown in Figures 3, 4. The quantitative analysis on the axial slices is summarized in Table 1. As mentioned earlier, five ROIs were selected on the pCT_r_ for calculating the SNU of the pCT_r_, original CBCT, enhanced CBCT and i-CBCT. The RMSD of the SNU between pCT_r_ and CBCT for fat and muscle ROIs were reduced by the proposed method from 109 to 13 HU and from 57 to 11 HU respectively, suggesting that the spatial uniformity of CBCT was markedly improved to a level close to that of pCT. The RMSD of the SNU between pCT_r_ and enhanced CBCT for fat and muscle ROIs were 14 and 7 HU, respectively, suggesting that the spatial uniformity of CBCT was improved to a level close to pCT to the same extent by both the proposed method and the existing method. In addition, the RMSD of the mean pixel values of ROI_m_ between pCT_r_ and CBCT for the fat and muscle ROIs were reduced by the proposed method from 216 to 11 HU and from 247 to 14 HU, respectively, suggesting that the pixel values of CBCT were improved substantially close to that of pCT. The RMSD of the mean pixel values of ROI_m_ between pCT_r_ and enhanced CBCT for fat and muscle ROIs were 10 and 10 HU, respectively, suggesting that the pixel values of CBCT were improved close to pCT to the same extent by both the proposed method and the existing method. The i-CBCTs are significantly better than the enhanced CBCTs on the PSNR (p < 0.01) and the SSIM (p < 0.01), suggesting that the noise reduction was performed more effectively and image quality become closer to pCT by the proposed method than by the existing method.

 

**Table 1:** Quantitative analysis of the image qualities for all the 20 cases. SNU is the difference between the maximum and the minimum mean pixel values among arbitrary positioned five square ROIs having 10-by-10 pixels. Under the column ROI_m_, the mean pixel value was shown, among the five ROIs, giving the maximum difference of the mean pixel values between pCT_r_ and original CBCT images (referred to as ROI_m_). Fat ROIs and muscle ROIs were used for patients 1-10 and 11-20, respectively. The RMSDs of the SNU and the mean pixel value of ROI_m_ between pCT_r_ and CBCT were calculated. PSNR and SSIM of CBCTs were measured based on pCT_r_ in the five ROIs used in calculation of SNU for each patient and resulting five values were averaged. pCT_r_: Registered planning computed tomography; original CBCT: Original cone beam computed tomography; enhanced CBCT: Enhanced cone beam computed tomography; i-CBCT: Improved cone beam computed tomography; SNU: Spatial non-uniformity; ROI: Region of interest; PSNR: Peak-signal-to-noise ratio; SSIM: Structural similarity index measure; RMSD: Root mean square differences.

	pCT_r_			original CBCT					enhanced CBCT					i-CBCT			
	SNU (HU)	ROI_m_ (HU)		SNU (HU)	ROI_m_ (HU)	PSNR	SSIM		SNU (HU)	ROI_m_ (HU)	PSNR	SSIM		SNU (HU)	ROI_m_ (HU)	PSNR	SSIM
Patient (Fat)																	
1	34	-118		135	-333	32.9	0.915		52	-132	48.5	0.930		54	-119	49.3	0.944
2	10	-106		94	-324	31.7	0.909		9	-109	49.5	0.937		5	-111	51.8	0.961
3	7	-100		69	-302	32.0	0.937		8	-102	50.5	0.954		20	-104	51.4	0.968
4	4	-103		160	-342	32.4	0.940		28	-103	50.1	0.959		24	-95	52.2	0.976
5	5	-110		91	-333	32.0	0.940		15	-118	51.0	0.960		19	-92	51.3	0.974
6	19	-114		131	-303	34.3	0.941		38	-121	49.5	0.955		29	-109	53.0	0.977
7	6	-121		194	-395	31.5	0.908		9	-119	48.1	0.912		18	-95	48.4	0.955
8	19	-100		88	-287	34.4	0.941		10	-101	48.8	0.945		23	-93	49.3	0.965
9	8	-120		103	-332	32.6	0.906		22	-137	47.7	0.922		24	-117	49.5	0.967
10	23	-120		89	-304	33.1	0.928		38	-140	49.3	0.944		17	-111	50.4	0.964
RMSD (Fat)				109	216				14	10				13	11		
Average (Fat)						32.7	0.926				49.3	0.942				50.6	0.965
Patient (Muscle)																	
11	29	34		98	-219	29.9	0.930		19	35	50.1	0.953		22	46	52.2	0.974
12	14	53		62	-209	29.5	0.932		16	51	50.0	0.948		18	30	49.6	0.973
13	10	48		76	-200	29.4	0.942		17	37	51.4	0.965		30	29	52.8	0.977
14	19	47		43	-124	32.7	0.954		20	43	51.7	0.966		27	34	52.9	0.976
15	11	54		70	-185	30.5	0.940		22	31	49.3	0.955		30	33	51.8	0.977
16	28	46		32	-163	30.7	0.943		18	43	50.9	0.962		16	53	52.1	0.974
17	16	41		112	-272	23.7	0.910		16	27	48.9	0.940		16	37	51.4	0.966
18	4	53		41	-194	29.2	0.937		3	51	50.6	0.957		9	38	49.4	0.975
19	24	40		99	-222	29.5	0.892		29	39	47.4	0.909		15	52	47.5	0.931
20	20	59		52	-183	30.0	0.917		30	47	48.6	0.934		13	54	52.7	0.971
RMSD (Muscle)				57	247				7	10				11	14		
Average (Muscle)						29.5	0.930				49.9	0.949				51.3	0.969
RMSD (Total)				87	232				11	10				12	13		
Average (Total)						31.1	0.928				49.6	0.945				50.9	0.967

**Figure 5:**
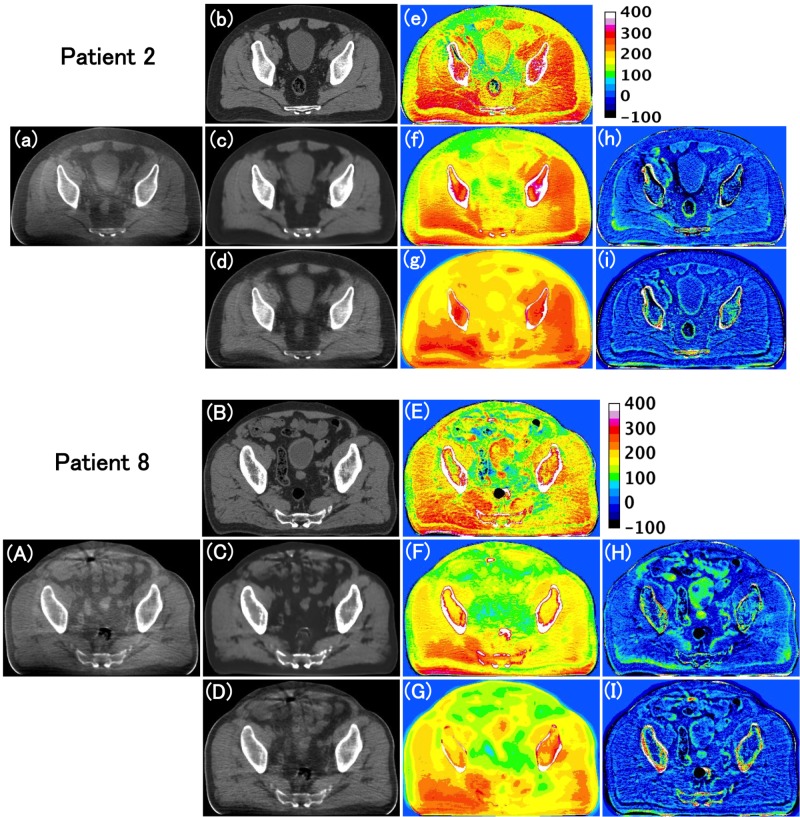
Comparison among original CBCT, pCTr, i-CBCT and enhanced CBCT for patient 2 and 8. (a), (b), (c) and (d) are original CBCT, pCT_r_, i-CBCT and enhanced CBCT, respectively; (e) pCT_r_ minus original CBCT; (f) i-CBCT minus original CBCT; (g) enhanced CBCT minus original CBCT; (h) pCT_r_ minus i-CBCT; (i) pCT_r_ minus enhanced CBCT for patient 2. (A) to (I) are for patient 8. original CBCT: Original cone beam computed tomography; pCTr; Registered planning computed tomography; i-CBCT: Improved cone beam computed tomography; enhanced-CBCT: Enhanced cone beam computed tomography.

The subtraction images to highlight the differences of pCT_r_ minus original CBCT are shown in Figure 5e, 5E, i-CBCT minus original CBCT are shown in Figure 5f, 5F, enhanced CBCT minus original CBCT are shown in Figure 5g, 5G, pCT_r_ minus i-CBCT are shown in Figure 5h, 5H and pCT_r_ minus enhanced CBCT are shown in Figure 5i, 5I. These differences in images shown in Figure 5 were calculated in the regions where registration errors between the original CBCT and the pCT were relatively large for patient 2 and 8. It was found that the structure difference of the rectum was clearly observed in Figure 5e, 5h but not in Figure 5f for the patient 2 and that of the bladder was clearly observed in Figure 5E, 5H but not in Figure 5F for the patient 8. This suggests that the proposed method successfully suppressed false structures from the pCT_r_ to appear on the i-CBCT. However, some structures (e.g., small intestines and right gluteus maximus muscle) on the original CBCT slice tended to be deformed or disappear on the corresponding i-CBCT slice in both cases.

## Discussion

A major advantage of the DCNN method may be the fast computation time. The training for the deep learning required a day, but once learning is done, i-CBCT was generated approximately in 20 seconds per patient using GPU. It is expected that the accuracy of the DCNN method will be further improved by using more training data. Although training time increases with larger training data, the size of the final model and the speed of processing a new test image will not be increased.

Table 1 showed that the RMSD of the SNU between the pCT_r_ and the original CBCT highly differed between fat (109) and muscle (57) HU. A possible interpretation may be that the proportion of fat near the body surface is greater than that of muscle, so the shading artifacts are more strongly observed in the fat region. In contrast, little differences were observed in the RMSD of the SNU between fat (13) and muscle (11) HU, and the RMSD of the mean pixel values of the ROI_m_ between fat (11) and muscle (14) HU for the i-CBCT. These findings may suggest that the spatial uniformity and the pixel values of the CBCT images were improved close to those of pCT regardless of the position and the anatomical structures on the CBCT images. Improvement of the PSNR and the SSIM on the enhanced CBCT than the original CBCT may be mainly due to the improvement of spatial uniformity and pixel value. On the other hand, improvement of the PSNR and the SSIM on the i-CBCT than the enhanced CBCT may be mainly due to the suppression of high-frequency artifact such as streaks.

Since pCT and CBCT images are typically acquired several days or weeks apart, differences in anatomical structure are often observed. We noticed that there was some residue deformation in the pairs of pCT_r_ and CBCT even after DIR, which might be due to the poor image quality of CBCT thus leading to inaccuracy of DIR. The registration errors of the training data may cause inaccuracy of the model since it may be trained to make false image predictions. The proposed method successfully suppressed false structures from the pCT_r_ to appear on the i-CBCT, even where the registration errors between CBCT and pCT were large. However, deformation and elimination of some structures (e.g., small intestines) were seen on the corresponding i-CBCT slice for some cases as shown in Figure 5, which may result in the registration error in IGRT. One potential approach to better preserve the anatomical structures on CBCT may be to improve the CBCT/pCT alignment of the training data (e.g., hybrid DIR [28]). Our future study will demonstrate the correlation between the alignment of the training data and the performance of the network to preserve the anatomical structures on CBCT. In addition, increasing learning data sequentially to construct a highly generalized network may also contribute to preserve the anatomical structures on CBCT.

In the proposed DCNN model, a 2D CBCT slice and a corresponding 2D pCT_r_ slice were used for learning. Employing multiple pairs of adjacent slices as input and output images in the model may be effective for making more accurate mapping from CBCT to corresponding pCT_r_. Alternatively, DCNN models can be trained in three different ways where the axial, sagittal or coronal slices of the 3D volume are separately used; subsequently, the three results may be averaged to produce final 3D i-CBCT. The evaluation of these extended approaches will be our future work.

All the training data used for learning the DCNN model in this study were obtained from a single pair of pCT and CBCT scanners. A DCNN model trained by a single-scanner training data may not be applicable to a test data acquired from a different scanner. In order to solve this issue, it may be necessary to standardize the CBCT images by preprocessing such as histogram-matching [29].

## Conclusions

We have developed a DCNN method for producing high-quality CBCT. The proposed method may be directly applied to the CBCT images acquired from a commercial CBCT scanner with high computational efficiency, allowing easy handling of large training data without sacrificing the speed of processing test data. Future work includes better preservation of the anatomical structures of CBCT during the improvement process, correlation between alignment of the training data and the performance of the network and further evaluation of the proposed method on larger data sets including other anatomical regions.
